# Using a Low-Sodium, High-Potassium Salt Substitute to Reduce Blood Pressure among Tibetans with High Blood Pressure: A Patient-Blinded Randomized Controlled Trial

**DOI:** 10.1371/journal.pone.0110131

**Published:** 2014-10-22

**Authors:** Xingshan Zhao, Xuejun Yin, Xian Li, Lijing L. Yan, Christopher T. Lam, Shenshen Li, Feng He, Wuxiang Xie, Ba Sang, Gesang Luobu, Liang Ke, Yangfeng Wu

**Affiliations:** 1 Department of Cardiology, Beijing Jishuitan Hospital, 4^th^ medical college of Peking University, Beijing, China; 2 The George Institute for Global Health at Peking University Health Science Center, Beijing, China,; 3 Department of Preventive Medicine, Feinberg School of Medicine, Northwestern University, Chicago, Illinois, United States of America; 4 Duke Global Health Institute, Duke University, Durham, North Carolina, United States of America; 5 Department of Epidemiology and Biostatistics, Peking University School of Public Health, Beijing, China; 6 Dangxiong People’s Hospital, Tibet, China; 7 Tibet Autonomous Region People’s Hospital, Tibet, China; Universtiy of Maryland Schoool of Medicine, United States of America

## Abstract

**Objectives:**

To evaluate the effects of a low-sodium and high-potassium salt-substitute on lowering blood pressure (BP) among Tibetans living at high altitude (4300 meters).

**Method:**

The study was a patient-blinded randomized controlled trial conducted between February and May 2009 in Dangxiong County, Tibetan Autonomous Region, China. A total of 282 Tibetans aged 40 or older with known hypertension (systolic BP≥140 mmHg) were recruited and randomized to intervention (salt-substitute, 65% sodium chloride, 25% potassium chloride and 10% magnesium sulfate) or control (100% sodium chloride) in a 1: 1 allocation ratio with three months’ supply. Primary outcome was defined as the change in BP levels measured from baseline to followed-up with an automated sphygmomanometer. Per protocol (PP) and intention to treat (ITT) analyses were conducted.

**Results:**

After the three months’ intervention period, the net reduction in SBP/DBP in the intervention group in comparison to the control group was −8.2/−3.4 mmHg (all p<0.05) in PP analysis, after adjusting for baseline BP and other variables. ITT analysis showed the net reduction in SBP/DBP at −7.6/−3.5 mmHg with multiple imputations (all p<0.05). Furthermore, the whole distribution of blood pressure showed an overall decline in SBP/DBP and the proportion of patients with BP under control (SBP/DBP<140 mmHg) was significantly higher in salt-substitute group in comparison to the regular salt group (19.2% vs. 8.8%, p = 0.027).

**Conclusion:**

Low sodium high potassium salt-substitute is effective in lowering both systolic and diastolic blood pressure and offers a simple, low-cost approach for hypertension control among Tibetans in China.

**Trial Registration:**

ClinicalTrials.gov NCT01429246

## Introduction

The WHO predicts that cardiovascular disease (CVD) will become the leading cause of Disability Adjust Life Years (DALYs) in 2020 [1] and more significantly, over 80% of this global burden will occur in low and middle income countries [2]. Further, hypertension accounts for nearly 45% of the global burden of cardiovascular morbidity and mortality [3]. Hypertension is one of the most common modifiable risk factor for CVD, with a prevalence of nearly 57% in adults 40 years and older in Tibet [4], two times as high as the 2002 China national rate [5]. This preponderance of hypertension has been strongly associated with an extreme burden of stroke in Tibetans. The Tibetan age-standardized stroke incidence was 450.4 per 100,000 persons and stroke mortality of 370.2 per 100,000 persons; both metrics were over four times the respective China national rates [6].

The high prevalence of hypertension in Tibetans is attributable to a very high level of daily dietary salt intake [7], [8]. In Tibetan adults the estimated dietary salt intake is nearly four to five times the WHO recommend amount of five grams daily [9]–[11], largely driven by the daily consumption of a traditional salty yak buttermilk tea [8], [9], [12], which has been reported to be as high as four liters per day in Tibetans [13]. Reduction in salt intake has been identified by the World Health Organization as a highly cost-effective strategy for cardiovascular disease prevention [14].

Low sodium salt substitute, as a low cost strategy to reduce sodium intake, has previously demonstrated marked reductions in systolic blood pressure among Han Chinese patients with high cardiovascular risk in the China Salt Substitute Study (CSSS). The magnitude of the effect was significantly associated with the baseline blood pressure of the CSSS cohort [15]. We set out to test the hypothesis that the low sodium dietary salt substitute could be more effective in reducing blood pressure in community hypertension programs in Tibetans living at high altitude, whose prevalence of hypertension as well as salt intake are markedly higher than Han Chinese [5], [16].

Of particularly note, the high altitude as the special regional living condition could actually cause significant difficulties to the local healthcare system in implementing hypertension prevention and control program as well as other health programs, such as lack of health care workers, remote and low access to health care services and medications, etc. Thus, the prevalence, treatment and control of hypertension in Tibet are very low [5]. This highlights the huge demand in developing strategies like salt substitute that are not only effective but can also be delivered through none-medical or para-medical health systems.

## Method

### Ethic statement

The trial was approved by the Ethics Committee of Peking University Health Science Center, Beijing, China (#IRB00001052-09003). All participants and the patriarchs of their families provided informed consent for the family. The protocol and CONSORT checklist are available as supporting information see Protocol S1 (English), Protocol S2 (Chinese) and Checklist S1.

### Design

A community based survey of hypertension was first conducted [5] and then all patients meeting inclusion and exclusion criteria were invited to the trial, with the intervention group receiving free salt substitute and the control group receiving free regular salt, in quantities sufficient for a complete household’s use during the three months’ study period between February 2009 and May 2009. The study was conducted according to principles of the Declaration of Helsinki and subsequent amendments and the study was registered at clinicaltrial.gov NCT01429246 in September 2011. This retrospective registration was due to our unawareness of the international requirements of prospective registration for such kind of trials at that time. However, we ensure honestly that the delay of registration has little bearing on the quality of science and ethics of this study.

### Study participantsand settings

Local residents aged 40 years and above from two townships (Yangbajing and Gongtangxiang) in Dangxiong County in Tibet Autonomous Region, China, were first invited to a hypertension screening, then those having measured systolic blood pressure ≥140 mmHg would be invited to the trial within 3 months. If the invitee’s systolic blood pressure was confirmed ≥140 mmHg again, regardless use of anti-hypertension medications, he/she would be invited to participate in this study. Of 287 patients who met our inclusion criteria, 282 were recruited after excluding 5 patients who met the exclusion criteria, including 1 patient who was too old and weak to travel, 1 patient who withdrew after inform consent, and 3 patients dropped by research staff due to their living far and arduous journey for follow up. The townships are located 90 kilometers northwest of Lhasa, with an average altitude of 4300 meters above the sea level.

The study was planned to enroll patients only in Yangbajing Township originally. However, there were not enough patients meeting our inclusion criteria and agreed to participate in the study there, so the recruitment had to be extended to the nearby township Gongtangxiang in order to achieve the recruitment target. But we reduced data to be collected at baseline in Gongtangxiang due to resource restriction. For details of differences in data collection between the two townships please refer to Table 1.

**Table 1:** Baseline characteristics of study participants by randomized group.

Characteristics		Total	Regular salt	Salt Substitute
(All subject)		(N = 282)	(N = 141)	(N = 141)
Age (years)		63.1 (11.2)	63.5 (11.3)	62.8 (11.1)
Sex (% female)		166 (58.9%)	81 (57.4%)	85 (60.3%)
SBP (mmHg)		176.9 (22.8)	177.6 (23.3)	176.1 (22.4)
DBP (mmHg)		104.5 (12.6)	105.8 (13.1)	103.2 (12.0)
Classification of hypertension^a^				
	Stage 1	56 (19.9%)	27 (19.1%)	29 (20.6%)
	Stage 2	226 (80.1%)	114 (80.9%)	112 (79.4%)
Antihypertensive medicine use in the past month (%)		132 (47.0%)	71 (50.7%)	61 (47.0%)
Average number of antihypertensive medicines taken		0.5 (0.5)	0.5 (0.5)	0.4 (0.5)
Body mass index (kg/m^2^)		23.6 (3.4)	23.6 (3.4)	23.7 (3.1)
**(Subjects in Yangbajing township only)**		**(N = 145)**	**(N = 72)**	**(N = 73)**
Education	Uneducated(0 year)	134 (92.4%)	70 (97.2%)	64 (87.6%)
	Primary school(1–5 years)	10 (6.9%)	2 (2.8%)	8 (11.0%)
	Middle school(6–9 years)	1 (0.7%)	0 (0.0%)	1 (1.4%)
Occupation	Herdsman	137 (94.5%)	71 (98.6%)	66 (90.4%)
	Others^b^	8 (5.5%)	1 (1.4%)	7 (9.6%)
Smoking history (%, yes)^c^		13 (9.0%)	7 (9.7%)	6 (8.2%)
Drinking history (%, yes)^d^		17 (11.7%)	9 (12.5%)	8 (11.0%)

All numbers shown are mean (±SD) unless otherwise noted as number (%).

All p-values comparing the two groups are larger than 0.05.

a. According to the American Heart Association, stage 1 hypertension was defined as 140≤SBP<159 and/or 90≤DBP<100; stage 2 hypertension as SBP≥160 and/or DBP≥100.

b. Other occupation includes farmer, doctor, self-employed and retired.

c. Participant who have smoke more than 20 packs in life or smoke at least one cigarette per day and last more than one year was regarded as having smoking history.

d. Participant who drinks at least once per week was regarded as having drinking history.

### Randomization

Stratified randomization by township, gender, and baseline systolic BP (<160 or ≥160 mmHg) was performed immediately after eligibility assessment, using a computer generated randomization list. A random number generator provides a treatment allocation identification number to each enrolled patient. This assignment was secured in a password protected encrypted digital registry. Treatment allocation was only blinded to participants through the use of indistinguishable containers; with only the study allocation number labeled. There was also no way to tell salt-substitute and regular salt by their physical appearance. The difference of taste between salt substitute and regular salt is minor. Our previous study among young adults indicated that about 70% −80% of testers were unable to identify the difference in taste in a triangle food taste test (Data unpublished). There was 1 patient from control group but no patient from intervention group in our study quoted “poor taste” as the reason for dropping out, indicating our blinding to patients were successful.

### Interventions

The anticipated allocation ratio between the intervention arm and control arm was set to 1:1. The intervention arm received a three months’ supply of salt substitute (65% sodium chloride, 25% potassium chloride, and 10% magnesium sulfate) [15], [17]. The control arm received regular salt with a 100% sodium chloride. The study salt was distributed free of charge to every household with sufficient amount to cover three months’ consumption for the whole household. Patients with preexisting anti-hypertensive medications were not directed to alter their prior regimen.

### Baseline measurements

Once the patients were confirmed eligible and the inform consent was obtained, the baseline measurements were conducted immediately. The screening, eligibility confirmation and baseline measurements were all conducted by trained research staff**,** between December 2008 and February 2009. Blood pressure was measured with three consecutive blood pressure measurements (with at least one minute’s rest between each measurement) from a seated subjects’ right arm in a quiet room. A previously validated electronic sphygmomanometer (OMRON HEM-759P, Dalian China) was used [18]. Weight and height were measured in a standardized way [5]. The personal information including age, sex, and current anti-hypertensive medication use were collected by structured questionnaire for all participants. Due to the resource constraints, other variables such as educational level, occupation, smoking history, and drinking history were obtained only from participants in Yangbajing at the screening survey [5].

### Follow up survey

After three months, all patients were asked to return for the measurements of blood pressure according to the same protocol used at baseline. We also collect data on anti-hypertension medication use during the whole study period for all participants.

### Outcomes

The primary outcomes were the changes in systolic and diastolic blood pressure from baseline to post-intervention over the three-month’ study period. The mean of the three systolic blood pressure measurements were used for analysis. The secondary outcomes were the proportion with hypertension under control (both systolic <140 and diastolic <90 mmHg) at post-intervention.

### Sample size

On the basis of our prior study, an estimated sample size of 230 total patients will provide 90% power, with a one-tail α = 0.05, and standard deviation of 13 mmHg to detect a 5.0 mmHg difference between two arms in change on systolic blood pressure at 3-month follow-up from baseline, with a 1:1 allocation of 115 to each treatment arm. We assumed a 20% of participants lost to follow up and thus a total of 287 participants should be recruited.

### Statistical Analysis

**Figure 1:**
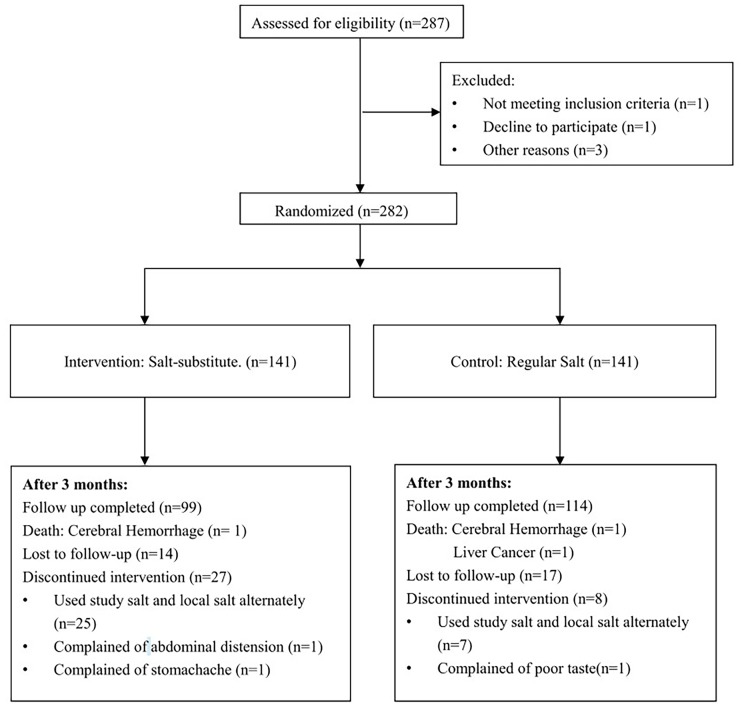
Trial Flow Chart.

Intention to treat (ITT) analyses was conducted and all eligible 282 subjects were included in the intention to treat analysis (**Figure 1**). We adopted multiple imputations (MI) approach to impute the missing values of SBP and DBP at 3 months since the baseline observations carried forward approach has recently been criticized for many limitations and may lead to serious bias [19], [20]. The imputation model included baseline systolic/diastolic blood pressure, age, sex, township, BMI and blood pressure lowering agents using at baseline. After excluding patients who were lost to follow-up, discontinued intervention, used outside salt, or died during the follow-up period, a total of 213 patients (99 in intervention arm, 114 in control arm) completed the follow up visit and were used in the per-protocol (PP) analysis. We used paired t-tests to test the difference in primary outcome between intervention and control, and we also used general linear model to adjust for variables used for randomization stratification (township, baseline systolic blood pressure, and gender) plus age, baseline BMI, and blood pressure lowering agents use. We were not able to adjust for other variables such as education, occupation, smoking and alcohol drinking since they were not collected in all participants. For the secondary outcomes, chi-square test was used. Primary outcome and secondary outcome were analyzed in both ITT and PP analysis. Statistical analyses were carried out using SAS 9.3 (SAS Inc., Cary, NC, USA).

## Result

### Baseline characteristics

The mean age of the 282 randomized subjects were 63.1 years at baseline, and 58.9% were females, 94.5% were herdsman, 92.7% reported never going to school, 9.0% were cigarettes smokers, 11.7% were alcohol drinkers, and 47.0% used anti-hypertension medication in the past month. Baseline BMI was 23.6 kg/m^2^. All these major baseline characteristics were comparable between the two randomized groups (all p-values >0.05) (**Table 1**).

### Effects on blood pressure reduction

**Figure 2:**
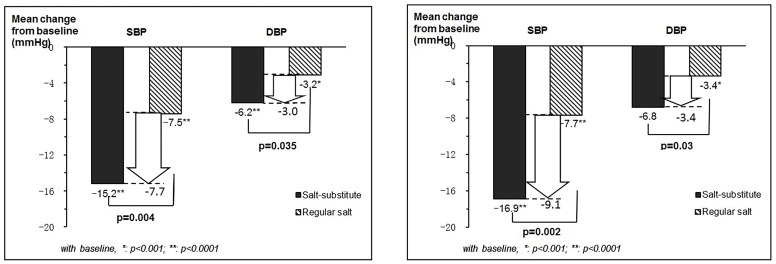
Unadjusted mean change of blood pressure levels at the 3-month follow-up from baseline for the salt substitute group and regular salt group in ITT and PP analysis. a: Intent-to-treat with multiple imputations approach, N = 282. b: Per-protocol analysis, N = 213.

Blood pressure was reduced in both groups and for both SBP and DBP at follow-up. However, the reduction was significantly larger in salt substitute treatment group for both SBP and DBP and in both ITT and PP analyses (Table 2). The intergroup unadjusted net reduction was −9.1 mmHg in systolic (95% CI: −3.2 to −15.0; p = 0.002) and −3.4 mmHg in diastolic blood pressure (95% CI: −0.3 to −6.4; p = 0.03) in PP analysis, but it was smaller at −7.7 mmHg in systolic (95% CI: −2.5 to −12.9; p = 0.004) and −3.0 mmHg in diastolic blood pressure (95% CI: −0.2 to −5.8; p = 0.035) in ITT analysis with multiple imputations approach (**Figure 2** **)**. After adjusting for baseline blood pressure, sex, age, township, baseline BMI and using blood pressure lowering agents, the net reduction was still significant, at −8.2/−3.4 mmHg (SBP/DBP) from PP analysis and at −7.6/−3.5 mmHg (SBP/DBP) from ITT analysis (Table 2).

**Table 2:** Blood pressure at baseline and follow-up, reduction of blood pressure in each group and net reduction of blood pressure in salt substitute group in comparison with regular salt group.

Variables	Groups		Baseline	Follow-up	△1^a^	△2^b^	p-value
***Per-protocol Analysis, (N = 213)***							
**SBP**	**Regular salt**	179.3 (23.6)		171.6 (27.1)	−7.7 (22.3)	−8.2 (2.9)	0.005
	**Salt substitute**	174.1 (20.6)		157.3 (25.7)	−16.9 (21.0)		
**DBP**	**Regular salt**	106.7 (13.5)		103.3 (14.1)	−3.4 (11.3)	−3.4 (1.5)	0.023
	**Salt substitute**	102.2 (12.0)		95.4 (14.8)	−6.8 (11.1)		
***Intent***-***to-treat Analysis with multiple imputations approach, (N = 282)^ c^***							
**SBP**	**Regular salt**	177.6 (23.3)		170.2 (26.8)	−7.5 (21.8)	−7.6 (2.5)	0.003
	**Salt substitute**	176.1 (22.4)		161.0 (27.0)	−15.2 (21.7)		
**DBP**	**Regular salt**	105.8 (13.1)		102.6 (13.8)	−3.2 (11.1)	−3.5 (1.4)	0.011
	**Salt substitute**	103.2 (12.0)		97.0 (14.5)	−6.2 (11.5)		

All numbers shown are mean (SD) except for the △2 that is shown in mean (SE).

a. Mean reduction of blood pressure in each group after intervention.

b. Net reduction of blood pressure in salt-substitute group in comparison with regular salt group, adjusting for baseline blood pressure, sex, age, township, baseline BMI and using blood pressure lowering agents.

c. impute 10 times.

### Effects on proportion of patients with blood pressure under control

**Figure 3:**
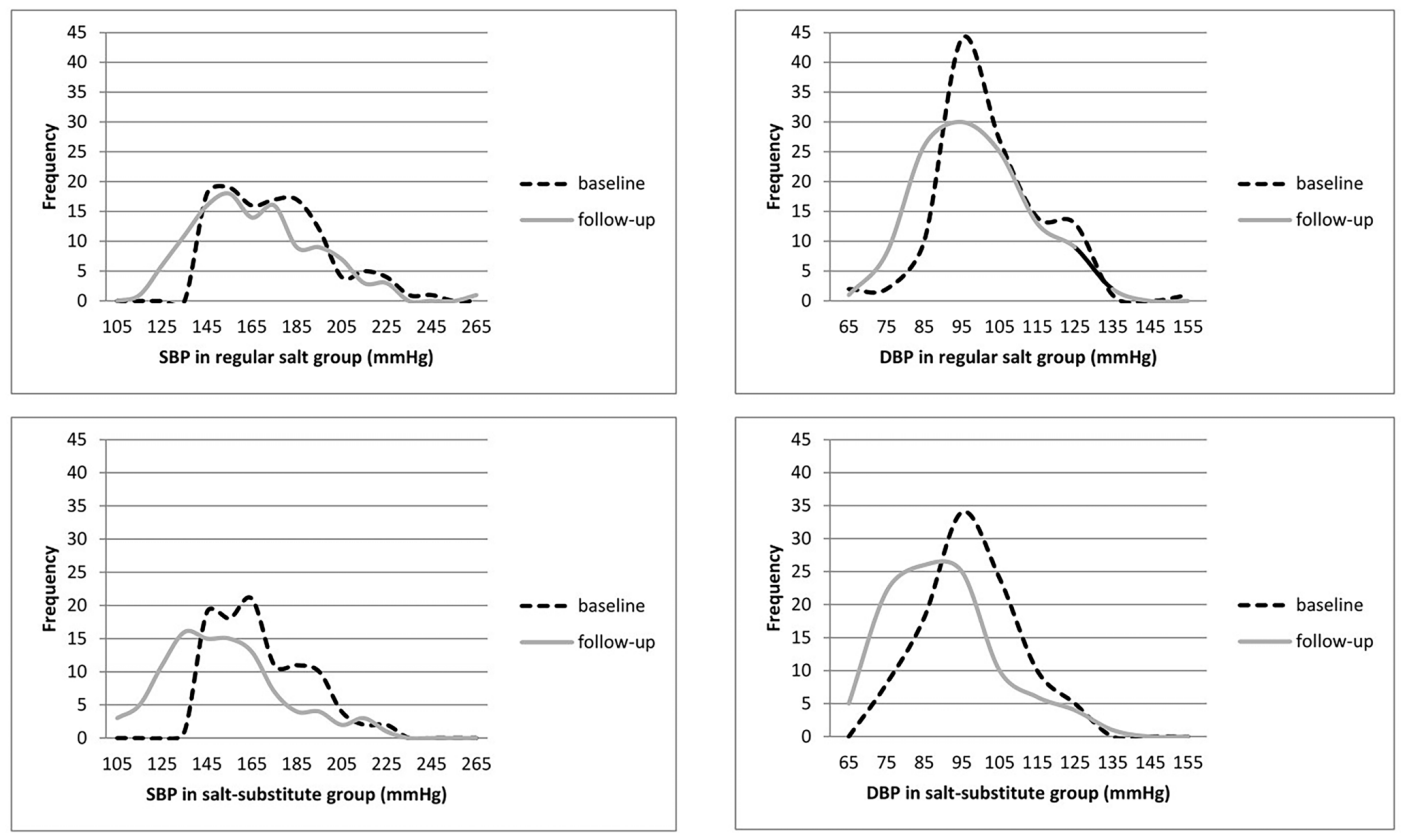
Distribution of Blood pressure at baseline and follow-up, per-protocol analysis.

All participants enrolled had systolic BP equal or greater than 140 mmHg at baseline. After three months’ intervention, PP analysis revealed that 8.8% in regular salt group and 19.2% in salt-substitute group had had their blood pressure well controlled (SBP/DBP<140/90 mmHg), (p<0.05). Although the peak remained around 160/95 mmHg (SBP/DBP), the overall blood pressure reduction in salt-substitute group was reflected by the dramatic shift of BP distribution to the left (Figure 3).

### Anti-hypertension medication use

At three months’ follow-up period, the proportion of patients taking antihypertensive medications in the past two weeks was lower in salt-substitute group than in the regular salt group (34.7% vs. 46.4%), however the difference was not significant (p = 0.081).

### Severe Adverse Events

Three people died during the study period, two in control arm and one in the intervention arm. Two of the three died from cerebral hemorrhage, one in each group, and the third death was due to liver cancer.

### Salt consumption estimation

Families located in the central of a group of families that participated in the study were selected by our staff on site for the convenience to distribute study salt, according to their geographic location. Our study staff delivered study salt to these families and then called for the study participants living nearby to come to pick up the study salt. This gave us the opportunity to measure the salt container at these families and ask the questions to help us estimate relatively accurate the amount of salt consumption in these families. For the selected household, we weighted the amount of study salt delivered to the household at baseline and the amount of remaining study salt at the follow-up to estimate average daily salt consumption per person. A total of 54 (27 in each group) families had complete and accurate salt consumption data and reported not using other salt (e.g. crude salt locally available, and/or salt bought from stores). The estimated average daily sodium chloride intake was 20.0±5.4 grams in the salt substitute group versus 26.9±8.1 grams in the regular salt group (p = 0.025). The additional potassium chloride intake is estimated at about 7.7±2.1 grams in the intervention group. Comparison between participants who did and did not have salt consumption data showed no significant difference in age, gender, blood pressure, education, smoking and alcohol drinking except for those with no data of salt consumption had a significantly lower proportion in use of anti-hypertensive drugs (48.5% vs. 64.8%, p = 0.022).

## Discussion

The present study demonstrated a significantly reduction in both SBP and DBP in salt-substitute group (by 7.6/3.5 mmHg in ITT analysis), which is expectedly even greater than what we found in China Salt Substitute Study (CSSS) conducted in rural Han Chinese, where a 5.4 mmHg reduction in SBP and no significant reduction in DBP were shown [15]. The result should be attributed most likely to higher blood pressure at baseline, but also markedly higher dietary salt intake, simpler diet and lower access to medical treatment in the Tibetan cohort in the present study in comparison to the CSSS’s Han Chinese cohort. Blood pressure reduction effect of an intervention is usually directly related to the baseline level of blood pressure, this is shown in many blood pressure lowering drug trials as well as non-drug trials such as DASH and CSSS [15], [21]–[24]. Compared to CSSS in which the baseline SBP/DBP was 159/93 mmHg, the baseline blood pressure in our study was much higher (177/105 mmHg). Furthermore, salt consumption for participants in our study was very high at about 27 grams per day but is about 15 grams in the CSSS study populations [15], [25]. The Tibetan diet is simpler, characterized by low consumption of fresh vegetables and fruits, and high meat and fat. There are evidences that a diet with rich fruits and vegetables could be beneficial to blood pressure lowering [26]–[28]. Compared with diets of CSSS study participants, the diet of Tibetan study participants was much simpler and lacked components that might be helpful in lowering blood pressure. At last, CSSS found that the blood pressure reduction effect of salt substitute was lower among patients with blood pressure lowering agents [15]. It is understandable that use of anti-hypertension medications would diminish the BP lowering effect from salt substitute through highly possible interactions. While 61% of study participants were having blood pressure medications in CSSS [15], it was only 47.0% in the present study. With smaller possible interaction with drugs, in comparison with CSSS, our study hence would have greater effect in lowering blood pressure. In this study, the significant reductions of both SBP and DBP were also found in the control arm receiving regular salt. These changes could be attributable to regression to the mean as well as the seasonal variations in blood pressure since the present study was conducted from February to May mirroring the expected temperature progression. This temperature associated variance phenomenon in BP has been previously reported by our group’s experience in the CSSS and many other prior reports [15], [29], [30].

In addition, we also found that blood pressure lowering agents use was lower in intervention group than in control group, though not statistically significant. This suggests the effect of salt substitute in lowering blood pressure demonstrated in the study would not be attributable to differential use of anti-hypertension medications. We believe that our observed effect would have an even higher magnitude if the use of anti-hypertension medications had been the same in both groups. The salt consumption data showed that the intervention group consumed 7 grams less sodium chloride and 7.7 grams additional potassium chloride on average per day, which, we believe, drove the blood pressure reduction significantly more in intervention group.

Due to the imbalanced lost to follow up between intervention and control in terms of quantity and quality, we choose multiple imputations rather than baseline observations carried forward approach to impute the missing values in our ITT analysis. In fact, our intervention group lost more patients than control group (42 vs. 27 patients). Comparison of those lost in intervention and control showed that patients lost in intervention group had higher blood pressure (180.9 vs. 170.3 mmHg in SBP), less use of blood pressure lowering agents (42% vs. 50%), less women (29.6% vs. 70.4%), younger age (64.0 vs. 65.3 years) and higher BMI (23.6 vs. 22.8 Kg/m^2^) at baseline. Although the results from baseline observations carried forward approach showed a smaller net reduction in both SBP (−5.9 mmHg) and DBP (−2.7 mmHg) and could be significantly underestimated the real net reduction, our results of both approaches showing a significant net reduction in SBP/DBP argue strongly for the validity of our study conclusion.

In order for population wide health intervention to be successful in a limited resource setting such as rural Tibet, the intervention and its delivery should not require a tremendous bandwidth of healthcare and financial capital. Furthermore, there is great importance for the cultural considerations in the design of the intervention, as approach of dietary restriction or outright elimination of the cultural dietary staple Tibetan yak buttermilk tea from study populations’ diet would have been a difficult intervention to implement. However, with the dietary salt substitute, the intake of sodium was successfully modified by dietary replacement of normal salt and population education. Thus, dietary salt substitute intervention presented in this study has the tremendous potential for benefits to public health as an simple, low-cost, and effective interventional strategy for ameliorating the burden of hypertension related maladies in both limited resource settings (i.e. Tibet) and developed countries.

The present study was limited by restricted financial resources available and austere environmental conditions (remote and rugged terrain at an elevation of 4300 m with serious oxygen deficit); which lead to a single follow-up at three months post randomization and precluded the ability to collect and analyze urinary sample to measure changes in sodium and potassium excretion. However, the present study was a randomized controlled trial design, which would minimize the biases and provide reliable observed effects yielding strong internal validity. Also due to the resource constraints, we were only able to collect baseline data on age, sex, SBP, use of blood pressure lowering agents and BMI. The other variables such as education, occupation, smoking and alcohol drinking were only collected for part of study participants and thus not included in the analysis for adjustment. However, the high comparability between the Intervention and Control in terms of these variables indicates that not having these variables adjusted would not significantly affect the internal validity. Thirdly, the salt consumption data collected in this study does not include sodium from natural foods and is only from a small fraction of the total study population. It should only be used for a rough estimation of sodium intake for local Tibetans. If possible, future studies in Tibet should consider use of 24-hour urine or dietary recalls to get more reliable data. Fourthly, our definition of hypertension excluded those with isolated diastolic hypertension and thus it should be cautious to extend our findings to isolated diastolic hypertension patients. Lastly, mean blood pressure in control group also dropped significantly after intervention. It was due to either seasonal blood pressure change or regression to the mean. The ‘white coat hypertension’ effect, if exist, should have been included in the regression to the mean. CSSS study had shown the similar seasonal blood pressure change where mean blood pressure reached the peak at the coldest season and the low ebb at the hottest season. However, our randomized parallel-controlled design well protected the validity of the study conclusion from the seasonal change and regression to the mean. Despite these limitations, this study was the first randomized control trial providing clear beneficial effects of salt substitute on blood pressure reduction in Tibet and area at high altitude.

In conclusion, this study confirmed the prior evidence of the blood pressure-lowering effects achieved with reduced sodium salt substitute in other trials. The excellent effect in lowering blood pressure achieved in this trial showed great potential of salt substitute in prevention and control of cardiovascular disease in this area and other similar settings in the world.

## Supporting Information

Protocol S1 **English translation of original protocol.** A study to develop simple interventions for control of hypertension in Tibet highland.
(DOCX)

Protocol S2 **Original protocol in Chinese.**
(DOC)

Checklist S1 **CONSORT 2010 checklist of information to include when reporting a randomized trial.**
(DOC)
