# Supplementary material for: Using a Low-Sodium, High-Potassium Salt Substitute to Reduce Blood Pressure among Tibetans with High Blood Pressure: A Patient-Blinded Randomized Controlled Trial
Source: PLoS One. 2014 Oct 22;9(10):e110131. doi: 10.1371/journal.pone.0110131 (PMC4206289; doi:10.1371/journal.pone.0110131)
Supplement: Protocol S1 — English translation of original protocol. A study to develop simple interventions for control of hypertension in Tibet highland. (DOCX) [file pone.0110131.s001.docx]

**A study to develop simple interventions for control of hypertension in Tibet highland**

**PROTOCOL**

CONTENT TABLE

BACKGROUND： 3

OBJECTIVES AND SPECIFIC AIMS: 5

STUDY DESIGN: 6

1. SURVEY ON PREVALENCE OF HYPERTENSION: 6

Including criteria: 6

Measurements: 7

*Height measurement:* 7

*Weight measurement:* 7

*Questionnaire:* 8

*Blood pressure measurement：* 8

*Sphygmomanometer calibration:* 9

2. SALT-SUBSTITUTE INTERVENTION: 9

Including criteria for salt substitute intervention: 9

Study design for salt substitute intervention: 10

*Stratification:* 10

*Registration:* 11

*Intervention:* 11

*Follow-up:* 12

*Sample size:* 12

EXPECTED RESULTS: 12

REFERENCE 14

# BACKGROUND：

Cardiovascular disease (CVD) severely endangers human health. The incidence of CVD in China is mounting up each year. Studies have shown that two third of cerebrovascular disease and one third of coronary heart disease (CHD) are attributed to high blood pressure. According to the national hypertension survey in 2002, among Tibetans, the prevalence of hypertension was 39.62% (46.64% in male and 35.74% in female), well above the national hypertension prevalence of 27.2%.[^1^](#_ENREF_1)

Pharmacotherapy was demonstrated to be an effective de-hypertension intervening measure for hypertensive patients or people at high risk of cardiovascular events. However, pharmacotherapy may not be preferred for those who are not diagnosed hypertension or high-risk patients of CVD. Though it accounts for the majority of blood pressure related events. Besides, the use of medication was affected by economic status and compliance of patients. Dietary intervention, as one of lifestyle intervention, has few side effects and barely influenced by economic status. If dietary intervention is feasible and widely implemented, it can reduce blood pressure and incidence of CVD at low cost. For patients have already received pharmacotherapy, dietary intervention can also bring additional benefit to them.

Observational epidemiological studies have shown a positive correlation between exceed sodium intake and blood pressure and a negative correlation between potassium intake and blood pressure.[^2-4^](#_ENREF_2) The most persuasive evidence on effect of sodium and potassium on blood pressure was from an observational study named INTERSALT that collected information from more than 10000 subjects from 52 centers worldwide to evaluate the relationship between urinary sodium/potassium excretion and blood pressure. Within and between 52 groups analysis,the excretion of Na+, K+ is significantly correlated with blood pressure. It is also correlated with the blood pressure increase which is related to aging or prevalence of hypertension..[^5^](#_ENREF_5)

However, in general population, it is difficult to limit salt intake, for changing their taste habit is diffecult. Therefore, low sodium and high potassium salt substitute (68% NaCl, 22% KCl and 10% Magnesium sulfate) is feasible to replace regular salt (100% NaCl) with salt substitute to reduce blood pressure. A previous study, China Salt Substitute Study (CSSS), has proved salt substitute can significantly reduce blood pressure. Ccompared to control arm, the systolic blood pressure (SBP) of intervention arm has dropped by 5.4 mmHg among Han Chinese.[^6^](#_ENREF_6)

High-altitude residents has formed special dietary habits due to special geography and climate condition. One survey conducted between 2001 to 2002 indicated that the diet structure of Tibetans was featured by high fat, high protein, high sodium and low fiber food. The average daily salt intake per person was 22±2.31g, well above that in Northern China, which was 12-18g.[^7^](#_ENREF_7) In Tibet, residents rely on home-cooking. Most dietary sodium are taken from salt added during food preparation, which provides a feasible way to reduce dietary sodium consumption by replacing regular salt with salt substitute.

Yangbajing locates in Dangxiong County, Tibet. The number of residents was 4812 among which 1297 are older than 40. There were three village councils: Laduogang, Jiabasha and Jiama and one township hospital. The living standard was fairly low. The average personal income was USD 316 in 2005. [^8^](#_ENREF_8)We took a vanguard for this study which indicated that the coverage of medical insurance was high but the area was short of doctors and medicines. Tibetan residents were not likely to visit doctor, for the drug type for hypertension was limited and the clinics were far away from their home.

# OBJECTIVES AND SPECIFIC AIMS:

The overall goal of this study is to estimate the prevalence and treatment of hypertension among Tibetans and develop blood pressure lowering interventions which are suitable for widespread implementation in the high-altitude area.

The specific aims include:

1. To investigate the prevalence and treatment of hypertension of adults older than 40 years old living in Yangbajing County of Tibet.

- To estimate the prevalence, awareness, treatment and control of hypertension as well as the accessibility to medical services for hypertensive patients among Tibetans.
- To investigate blood pressure-related lifestyle and dietary habit among Tibetans.

1. To develop simple, applicable and effective interventions to reduce blood pressure of Tibetans.

- To confirm the effect of low sodium and high potassium salt-substitute on reducing blood pressure among Tibetans.
- To evaluate the acceptability of salt substitute among Tibetans.
- To explore the feasibility of using salt substitute plus low dose diuretics in reducing blood pressure for Tibetan hypertensive patients.

# STUDY DESIGN:

This study consists of two parts: one is a cross-sectional survey on prevalence of hypertension; the other is a randomized controlled trial to explore the effect of salt-substitute on reducing blood pressure among Tibetans.

## 1. SURVEY ON PREVALENCE OF HYPERTENSION:

### Inclusion criteria:

Doctors from Yangbajing township clinics will visit every Tibetan resident who is age-eligible (≥40 years old) and living in Yangbajing Township. Questionnaire survey will be conducted followed by blood pressure measurement.

Individuals will be invited to participate if they meet **ALL** the following criteria:

- Age≥40 years old;
- Being Tibetans;
- Permanent residents of Yangbajing Township.

Participants will be excluded if they are living with severe somatoform or mental disorders so that they are unable to participate in this survey.

### Measurements:

Physical examination was conducted for each individual meeting inclusion and exclusion criteria, and personal characteristics were collected through survey questionnaire.

#### *Height measurement:*

International standard of height measurement was adopted. A stadiometer was fixed against a flat wall and perpendicular to the floor. We will ask each patient to stand upright on floor with shoes and hats removed, looking straight ahead, putting arms at sides, and keep the shoulders, buttocks, and heels touching to the wall. After that, we will use a triangle ruler to form a right angle with the wall and make another triangle side firmly touch the crown of the subject’s head, read the record from the alignment and accurately record the height to 0.1 cm.

#### *Weight measurement:*

We will use spring scales to measure each subject’s weight by international standard of weight measurement. Scales will be calibrated before each weighing. In light of cold weather, subjects will not be asked to undress, only need to take off hats and shoes and on an empty stomach and bladder. Subjects will stand on the center of platform and distribute weight evenly on both feet. Investigators will record the weight to 0.1kg as rough weight when the pointer of scale is stable. In order to require exact weight of body, we will weight several kinds of common dresses of Tibetans and record them to 0.1kg as clothes weight. For each participant, the body weight will be calculated as rough weight minus clothes weight.

#### *Questionnaire:*

We will conduct survey for all participants through survey questionnaire (see appendix 1) that includes five parts: (1) basic personal characteristics: age, sex, ethic, and occupation; (2) lifestyle information: tobacco use, tea drinking, and alcohol use; (3) diet structure: staple, meat, and vegetable intake frequency; (4) medical insurance level; (5) hypertension and its relative medical history: the prevalence of awareness and treatment of hypertension, their knowledge on anti-hypertensive; their compliance of high blood pressure medications, and the history of using Tibetan medicine. Professionals who can speak both Chinese Mandarin and Tibetan will ask questions related to Tibetan medicine.

#### *Blood pressure measurement：*

Blood pressure will be measured for each participant by automated blood pressure monitoring device (OMRON HEM-759P, Dalian China). We are going to create favorable conditions and sustainable measuring environment for blood pressure measurement. The measurement site needs to be quiet and bright, providing room for participants to take rest. Participants need to take at least ten minutes rest and empty their bladder before having their blood pressure measured. Before coming to the examination, participants will be asked to abstain from drinking tea or coffee, smoking, and heavy exercise for at least 15 minutes before the measurement. Each participant will be in a sitting position with feet resting on the floor and arm resting on the desk. The cuff is placed on the right arm and its bottom edge is about 2.5 cm above the antecubital fossa. Investigators will correct cuff size for the arm circumference and make the tubes from the cuff align with participant’s brachial artery. Blood pressure will be measured and recorded with twice measurements (with at least waiting 30 seconds to allow for the cuff deflated completely).

#### *Sphygmomanometer calibration:*

The barometric pressure in Tibet is too low for normal operation of electronic sphygmomanometer. We have conducted a study to valid electronic sphygmomanometer (OMRON HEM-759P, Dalian China) with mercury sphygmomanometer. The study on 129 participants showed asignificant correlation between the measurements of electronic sphygmomanometer and mercury sphygmomanometer. The mean deviation for systolic blood pressure (SBP) was 5 mmHg and no deviation for diastolic blood pressure (DBP) by which the reading from electronic sphygmomanometer would be calibrated.[^9^](#_ENREF_9)

## 2. SALT-SUBSTITUTE TRIAL:

### Inclusion criteria for salt substitute intervention:

Village doctors will visit potential hypertensive patients (SBP≧140 mmHg or SBP ≧90) screened out in the survey three months later to verify their blood pressure. Patients meeting inclusion and exclusion criteria can take part in this study after signing the informed consent (Appendix 3).

If more than two people in one household meet our inclusion and exclusion criteria, only one of them will be randomly selected.

Inclusion criteria of salt substitute intervention:

1. Age≥40 years old; AND
2. SBP≥140 mmHg and/or DBP≧90 mmHg.

Exclusion criteria:

Patients will be declined if they or their families have history of severe renal impairment, gout or they were recommended by doctors that they should not use salt-substitute. Besides, if participants could not comply with randomization, they would not be enrolled.

Anti-hypertensive medication is not considered as exclusion criteria and patients with preexisting anti-hypertensive medications were not directed to alter their prior regimen.

### Study design for salt substitute trial:

This study is a subject-blinded randomized control trial with two-stage intervention for six months, the first three months for first-stage intervention and the last three months for the second-stage. (Figure 1) It is not possible to visually identify which type of salt the salt container contain from the appearance and size of the granules.

#### *Stratification:*

Patients will be divided into two stratus by baseline blood pressure: 140≤SBP<160 mmHg and SBP>160 mmHg. In each strata, patients will be randomly assigned to receive salt substitute (intervention group) or regular salt (control group). Randomization was done immediately after eligibility assessment, using a computer generated randomization list; with a random number generator providing a treatment allocation ID number to each enrolled patient.

#### *Registration:*

The household ID, subject ID, name, family members, randomized group of enrolled subjects and the number written on their distributed salt package will be registered by investigators.

#### *Intervention:*

The first stage: The intervention group will receive a three months’ supply of salt substitute (65% sodium chloride, 25% potassium chloride, and 10% magnesium sulfate). The control group will receive regular salt with a 100% sodium chloride. The study salt will be distributed free of charge to every household with sufficient amount to cover three months’ consumption for the whole household in cooking meals, making yak tea[^10^](#_ENREF_10) or bacon, or being add as table salt etc. The amount of salt delivered for household will be calculated on basis of estimated 30g per person per day and the supply of salt will be adequate for more than three months. The containers for salt substitute and regular salt will be identical and have unique number on them. Meanwhile, every patient will receive health education on prevention and treatment for hypertension as well as salt reduction.

The second stage: After three-month intervention of using salt substitute, intervention group and control group will continue to receive salt substitute and regular salt respectively. For whose SBP still higher than 140 mmHg or DBP higher than 90 mmHg at the first-stage evaluation visit, they will receive low-dose diuretic (hydrochlorothiazide, 6.25mg/day) in the second stage intervention that will last another three month.

#### *Follow-up:*

At one-month after intervention, investigators randomly select samples to visit mainly for the purpose of investigating salt consumption information in order to know their acceptance of salt substitute and regular salt. At three-month after intervention, investigators will visit every subject, measure blood pressure and weight amount of salt-consumption, register each subject’s household ID, name, total amount of salt consumption of the whole household for three month and subject’s blood pressure. For subjects whose blood pressure have not been controlled, we will offer them low dose of diuretics for enhancing treatment. The salt for the next three months intervention will be distributed to each household participated in this visit.

#### *Sample size:*

On the basis of our prior studies, an estimated sample size of 230 total patients will provide 90% power, with a one-tail α = 0.05, and σ = 13 mmHg to detect a 5.0 mmHg difference in systolic blood pressure, with a 1:1 allocation of 115 to each arm.

N=230

α=0.05 (one side)；β=0.10；σ=13mmHg; δ=5mmHg.

# EXPECTED RESULTS:

1. The prevalence, awareness, treatment and control of hypertension among Tibetans older than 40 living in Yangbajing County.
2. A significant reduction on blood pressure and improvement of hypertension treatment for hypertensive patients in intervention arm.
3. Significant difference of blood pressure reduction between intervention arm and control arm.
4. The feasibility and effect of salt-substitute plus low-dose diuretics on controlling blood pressure for hypertensive patients living in high-altitude area.

# REFERENCE

1. Wu Y, Gao R, Kong L, Yang X, Huxley R, Li L, et al. Prevalence, awareness, treatment, and control of hypertension in China: data from the China National Nutrition and Health Survey 2002. Circulation. 2008; **118**(25): 2679-86.

2. He J, Whelton PK. Potassium, Blood Pressure, and Cardiovascular Disease: An Epidemiologic Perspective. Cardiology in Review. 1997; **5**(5): 255-60.

3. Elliott P. Observational studies of salt and blood pressure. Hypertension. 1991; **17**(1 Suppl): I3-I-8.

4. de Wardener HE, MacGregor GA. Sodium and blood pressure. Current Opinion in Cardiology. 2002; **17**(4): 360-7.

5. Elliott P, Stamler R, Stamler J, Nichols R, Dayer AR, Kesteloot H, et al. Intersalt Revisited: Further Analyses Of 24 Hour Sodium Excretion And Blood Pressure Within And Across Populations. BMJ: British Medical Journal. 1996; **312**(7041): 1249-53.

6. Group CSSSC. Salt substitution: a low-cost strategy for blood pressure control among rural Chinese. A randomized, controlled trial. J Hypertens. 2007; **25**(10): 2011-8.

7. Liu L, Mizushima S, Ikeda K, Nara Y, Yamori Y, Liu L, et al. Ethnic and Environmental Differences in Various Markers of Dietary Intake and Blood Pressure among Chinese Han and Three Other Minority Peoples of China: Results from the WHO Cardiovascular Diseases and Alimentary Comparison(CARDIAC) Study. Hypertension Research. 2001; **24**(3): 315-22.

8. Wang L, editor. Nutrition and Health Status in Chinese People. Beijing, China: People's Publishing House; 2005.

9. Yangfeng LSZXBSHFKLYLW. Validation of electronic sphygmomanometers against mercury sphygmomanometers at high altitude in Tibet International Journal of Cardiology. 2009; **137**: S19.

10. Sang L. Component Analysis on Northern Tibet Yak Meat and Research on Its Nutrition Quality [J]. Journal of Anhui Agricultural Sciences. 2009; **29**.

**Figure 1: Flowchart of salt substitute intervention**

Second-stage

Salt substitute plus diuretic

First-stage

The first follow-up

(3 months)

Regular salt

(Control arm)

Salt substitute

(Intervention arm)

The second follow-up

(6 months)

Salt substitute

Regular salt

Regular salt plus diuretic

Randomization

Registration
