# Supplementary material for: Using a Low-Sodium, High-Potassium Salt Substitute to Reduce Blood Pressure among Tibetans with High Blood Pressure: A Patient-Blinded Randomized Controlled Trial
Source: PLoS One. 2014 Oct 22;9(10):e110131. doi: 10.1371/journal.pone.0110131 (PMC4206289; doi:10.1371/journal.pone.0110131)
Supplement: Protocol S2 — Original protocol in Chinese. (DOC) [file pone.0110131.s002.doc]

**西藏高原地区高血压防治简易方案研究**

**研究方案**

# 研究背景

心血管疾病是危害人类健康的主要疾病。我国的心血管病发病率有逐年上升的趋势。研究已证明，大约2/3脑血管疾病和1/2的冠心病负担是由于不理想的血压情况造成的。2002年全国高血压普查中，对西藏地区的高血压调查中发现，当地居民高血压的患病率为39.62%，其中男性为46.64%，女性为35.74%，远高于全国高血压的患病率27.2%。

对确定的患高血压的人群或心血管病高危人群来说，基于药物降压措施已经被证实为降低心血管疾病的有效方法。但是，许多血压相关事件发生在不属于上述两种情况的人群，对他们通常不会进行药物干预。而且，药物的使用情况受经济和患者依从性的影响。膳食干预作为生活方式干预的一种，没有或较少有副作用，且较少受到经济条件的影响。如果可行且广泛实施，将会使人群血压降低，预计也会实质性地降低心血管疾病的发生率，同时花费也少。另外，对那些接受药物治疗的病人，饮食降压措施仍然会有额外的益处。

观察性流行病学研究证实更多的钠摄入和血压之间存在正相关。与此相应，钾摄入水平和血压之间存在负相关。关于钠和钾摄入与血压水平关系的最有说服力的观察性资料来自国际盐和血压研究（INTERSALT）。这项现况研究收集了全世界52个中心超过10000名对象的数据，以评价血压与钠和钾的排泄在群体内和群体间的关系。在52个研究群体内及群体间，24小时尿阳离子排出与平均收缩压和平均舒张压有关，与随着年龄的血压增加以及高血压的患病率也有关。

但是，在一般人群中，单纯的限制盐的摄入量即让人们改变他们饮食的口味，是难以做到的。低钠高钾代用盐，是一种68% 氯化钠, 22% 氯化钾, 10% 七水硫酸镁组成的混合物。用代用盐代替单纯NaCl的普通盐，是减少钠离子摄入，从而降低血压的可行方法。中国代用盐研究（China Salt Substitute Study）已经清楚的证明使用代用盐能明显的降低病人的血压。在一年的干预期后，干预组（使用代用盐）较对照组（使用普通食盐）的收缩压降低了5.4 mmHg。

高原居民，由于其居住地特殊的地理条件和气候条件，形成特殊的饮食习惯。在2001-2002年的一项调查中显示，拉萨市藏族人群的膳食结构以高脂肪、高蛋白、高钠和低纤维素的食物为主；其中每人每日钠盐的摄入量为22±2.31g，高于我国北方人的平均食盐量（12-18g）。而且，当地居民大部分食物是在家中制作，膳食中的钠多数是从烹饪时加入的盐中获得，因此考虑，在西藏地区使用代用盐降低高血压是可行的。

羊八井镇位于西藏拉萨市当雄县。**全镇人口为4812人**，40岁以上人口为1297人。共有三个村委会：拉多岗、贾巴沙和甲玛。当地有一个镇卫生院。当地居民的生活水平较低，人均收入为2558.93元（2005年）。在前期走访中发现，当地医疗保险制度覆盖率高，但是面临缺医少药的局面，仅有很少种类降压药品。且当地居民存在距离医疗机构远，不愿轻易就医的问题。

# 研究目标

了解西藏地区藏族居民高血压的患病情况和治疗情况，并提出适宜高原地区推广的降压方案。具体研究目的包括：

1. 调查西藏羊八井地区40岁以上居民的高血压患病情况以及治疗情况
   - 了解藏族居民的高血压患病率、知晓率、治疗率和控制率以及高血压患者的就医情况
   - 了解藏族居民与高血压相关的生活方式和饮食方式的调查
2. 提出适宜藏族居民高血压患者的简单有效的降压方案
   - 确定低钠高钾代用盐（代用盐）在藏民中是否能作为控制高血压的一项有效措施及其有效性
   - 了解藏族居民对代用盐的可接受性
   - 探讨代用盐+小剂量利尿剂是否可以作为藏族高血压患者简单有效的降压方案

# 研究设计

**本研究根据研究目的可分为两个部分：一是对西藏羊八井地区藏族居民的高血压流行情况进行普查；二是在当地高血压患者中开展高钾低钠盐+小剂量利尿剂的随机对照试验**

**1、高血压普查及相关情况调查：**

1.1入排标准：

三个乡卫生所或卫生服务点的医生将对所有在调查期间在羊八井镇居住的40岁以上**藏族居民**进行入户的问卷调查（先于血压测量进行）、血压普查，并登记结果。

入选标准：年龄≥40岁；藏族；为羊八井镇常住居民

排除标准：患有严重的躯体或精神疾病，无法参加调查。

1.2 对所有参与普查居民，同时进行体格检查，测量身高和体重，填写《高血压普查信息记录表》（附表一）。

身高的测量：采用国际化标准化方法测量。用边长100cm的皮尺钉在光滑的与地面垂直的墙上，使皮尺与地面垂直。请被检者脱去鞋帽，光脚站立，脚跟并紧，脚后跟、臀部、肩和头贴近墙壁，双眼正视前方。用直角三角尺的一条直角边紧挨头顶骨上方，另一条直角边紧贴墙壁。被检者离去后读取三角尺直角顶点处的读数，读数以cm为单位。

体重的测量：采用国际化标准化方法测量。使用弹簧式体重计，每次测量前校正零点。考虑到当地气候寒冷，被检者可以不必脱掉衣物，只需摘掉鞋帽、空腹并排空膀胱，站于体重计盘中央。待指针停稳后记下读数（精确到0.1Kg）。此计数为粗重。将藏民常见不同服装称重并记数（精确到0.1Kg），此计数为服装重量。居民的体重=粗重-所着衣物的重量

1.3对所有参与普查的居民，进行高血压相关情况的调查，填写《高血压普查信息记录表》（附表一）。调查内容分为三部分：①居民基本信息：年龄、性别、民族、职业等；②生活方式调查：吸烟情况、饮茶、饮酒情况；③膳食结构调查：主食、肉类和蔬菜的摄入情况，盐的摄入情况；④医疗保障情况调查；⑤高血压及相关病史情况的调查：高血压的知晓及治疗情况，是否接受了降压药物治疗，药物治疗依从性，是否接受过藏医/藏药治疗，藏医药治疗内容等调查将由双语（汉语/藏语）兼懂的专业人员进行询问。

1.4对所有参与普查的居民，进行血压的测量：使用电子血压计测量，型号为：欧姆龙电子血压计HEM-759P。测量要求时要保证良好的测压环境和稳定的测压条件，室内保持安静明亮，要备有让测压者坐下休息的条件。测压前，受检者需安静休息10分钟，精神放松，避免用力，要排空膀胱，受检前15分钟停止吸烟，测血压前避免饮用茶、咖啡类饮料。测量时，受检者取座位，双足平放在地面上，右胳膊放在桌面上，支撑应舒适，手掌向上，袖带的下缘放置在肘关节前自然皱折上方约2.5cm处，袖带要平整舒适地绑在右上臂上，不能太松或太紧，使充气的气囊中心正好位于肱动脉的部位。连接血压计和袖带，开始测量血压：第一遍测压结束后，记录数值；待袖带完全放气后等待三十秒，再测第二遍，并做好记录，取下袖带整理好，并妥善保存血压计。

1.5 高原血压计的测量校正：由于高原地区是低气压环境，气压已低于电子血压计的正常工作气压。所以在研究开始前用三通管将汞柱血压计和电子血压计并联，对129人的血压进行测量，用汞柱血压计校正电子血压计。统计结果显示，电子血压计的测量值与汞柱血压计的测量值高度相关。收缩压平均值相差5mmHg, 舒张压平均值没有显著差异。电子血压计的矫正方法是，收缩压减去5mmHg，舒张压不变。

**2、干预研究：**

1.1 **研究入选**：

普查研究发现的高血压患者，医生将于3个月后入户对其血压进行再次测量，所有两次测量血压结果收缩压均≥140mmHg或舒张压均≥90mmHg的高血压患者，若符合干预研究的入排标准，经知情同意（附件三），可自愿进入干预研究。以户为单位，每户随机选取一人为观察对象，最少入选230名（即230户）居民。

入选标准：1. 年龄≥40岁

2. 收缩压≥140mmHg或/和舒张压≥90mmHg

排除标准：如果待选对象及其家属正在服用保钾药物，或确定有明确的肾脏疾患或痛风病史、按其负责医师的意见不能使用代用盐的话，此待选对象将不入选本研究。另外，如果有任何原因待选对象一定要使用代用盐（或普通盐）或者不能使用代用盐（或普通盐），也不能入选本研究。

是否服用降压药不作为入排标准。在研究期间，病人的其他治疗将保持不变。

**1.2研究设计：**

本研究为两阶段随机对照单盲试验，试验用盐（代用盐和普通盐）将采用统一包装，参与研究对象无法从外包装上识别其所使用盐的种类。研究将持续6个月，第一阶段持续3个月，第二阶段持续3个月。其中随访2次，共纳入羊八井镇的230名高血压患者。

**分组**：将按照高血压患者性别和其收缩压水平分（140-160mmHg、160以上）分为四层。每层居民被随机分为干预组（代用盐组）和对照组（普通盐组），确保每个分层中的患者被平均分到干预组和对照组。随机分配过程将由一台计算机通过随机化程序在现场完成。

**登记**：对当地参与研究的个体进行登记。登记内容包括：户号、观察对象ID号及姓名、人口数、随机组别和研究用盐的编号。

**干预**：

第一阶段：干预组的居民将接受低钠高钾代用盐（68% 氯化钠, 22% 氯化钾, 10% 七水硫酸镁）；同时对照组居民将使用普通盐 （100%氯化钠）。研究者将为每一名受试对象及全体家庭成员提供足够的代用盐或普通盐，以满足其所有的家庭需求（即烹饪、煮茶、做腌肉、进餐时自行添加以及其他所有用途）。估计每一人每天消耗约30克食盐。一次提供3个月量的代用盐或普通盐，让他们可以用到第二阶段。代用盐和普通盐的包装相同，用编号进行标记。同时所有患者将接受高血压防治和减少用盐的健康教育。

第二阶段：在单纯使用代用盐干预3个月后，干预组和对照组分别继续给与代用盐或普通盐。其中对于那些收缩压仍然大于140mmHg和/或舒张压仍大于90mmHg的患者，给与小剂量的利尿剂双氢克尿塞6.25mg/天。第二阶段干预再持续3个月。

**随访**：研究开始后1个月，工作人员将随机抽取部分受试对象进行随访，观察其用盐状况，以估计受试对象对普通盐和代用盐的接受情况。在研究第3个月末，工作人员将随访所有受试对象，测量血压和用盐量，并填写随访情况记录表，内容包括户号、观察对象ID号及姓名、三个月用盐量及观察对象血压。对血压未降到140mmHg/90mmHg以下的患者，将给与小剂量利尿剂进行加强治疗。在此次随访中还将发放后3个月的盐给研究家庭。

登记

代用盐（干预组）

n=115

普通盐（对照组）

n=115

随机化

随访（终点为第6个月）

**研究流程**

代用盐+利尿剂

继续代用盐

继续普通盐

普通盐+利尿剂

第三个月末随访

**1.3样本量分析：**

样本量为230人，随机分配至干预和对照组，每组为115名病人。此样本量是根据单侧检验显著性水平为0.05，检验效能为0.90，假设从基线至6个月随访两组平均收缩压变化的差值在5.0 mmHg的条件进行计算的。（数据来自于北京地区代用盐研究）

α=0.05(单侧)；β=0.10；σ=13mmHg; δ=5mmHg;

N=230

# 预期研究结果

1. 西藏羊八井地区40岁以上居民的高血压患病率、知晓率、治疗率和控制率
2. 干预组的高血压患者的血压降低，且具有统计学意义。高血压病情有改善
3. 干预组患者与对照组患者相比，血压降低。
4. 代用盐+小剂量利尿剂能有效控制高原地区高血压患者的血压，为以后的研究提供依据。

附表一： **西藏高原地区高血压防治简易方案研究**

《高血压普查信息记录表》

填表说明：

1. ID包括三部分，第一部分为村\镇的编号，第二部分为组的编号，第三部分为居民的编号，按普查顺序从0001开始编号。
2. 所有空格均从最后一位填起，且必须填满。空位时补“0”，不详时填“9”

**第一部分：ID号和基本情况**

| 1.1．ID: -- | | 1.2．日期(年/月/日): 200年月日 | |
| --- | --- | --- | --- |
| 1.3．户主姓名：___________ | 1.4．户编号： | | 1.5．家庭常住人口数： |
| 1.6．藏语姓名___________ | | 1.7．汉语姓名__________ | |
| 1.8．性别: 1=男 2=女  | | 1.9．出生年月: 年月 | |

第二部分：家庭情况调查表（只询问户主）

| 2.1. 您的家庭目前持有的牲畜数量 牛 | 头 | | |
| --- | --- | --- | --- |
| 羊 | 头 | | |
| 马 | 头 | | |
| 2.2.您家的房子共有多少间 | 间 | | |
| 2.3. 您的家庭目前持有下列物品的数量 电视 |  | | |
| 货车或拖拉机 |  | | |
| 轿车 |  | | |
| 摩托车 |  | | |
| 2.4. 您家庭的每月食盐量是 | | 克 |  |

**第三部分：社会经济状况**

| 3.1. 您的文化程度是: 0=未上学 1=小学 2=初中 3=高中或中专 4=大专以上 | |  |  |
| --- | --- | --- | --- |
| 3.2. 您现在的职业（按所从事实际工作的性质）属于下列哪类？ |  | | |
| 01=工人 02=农民 03=牧民 04=教师 05=医生 06=专业技术人员 07=政府工作人员 08=服务人员 09＝个体经营者 10=干部 11=离退休人员 12=家务 13=其他 | | | |

| 3.3.1 您每天平均睡眠的时间有多久? | .小时 |
| --- | --- |
| 3.3.2 您每天平均看电视的时间有多久? | .小时 |
| 3.3.3 在夏秋两季您每天平均放牧的时间有多久? | .小时 |
| 3.3.4 在冬春两季您每天平均放牧的时间有多久? | .小时 |
| 3.4. 您现在工作时体力活动强度和10年前相比:  1=减轻 2=不变 3= 加重 |  |

**第四部分: 生活方式和行为**

| 4.1.您曾吸过烟吗? ( “是”指一生中至少吸过20包，或每日至少吸1支且连续吸至少一年) 0＝否(**转问4.2. 饮酒**) 1=是 |  |
| --- | --- |
| 4.2.您多大年龄开始有规律吸烟的? | 岁 |
| 4.3.您现在或最近吸烟吗? 0=否 1=是**（转问4.3.1）** |  |

| 4.3.1.您多大年龄停止吸烟的？ | 岁 |
| --- | --- |
| 4.3.2.为什么停止？1＝疾病 2＝其它 |  |

| 4.4.您现在每天吸多少支香烟? | 支/天 | | | | |
| --- | --- | --- | --- | --- | --- |
| 4.5.您在整个吸烟期间平均每天吸多少支香烟? | 支/天 | | | | |
| 4.6.您曾饮酒吗?（至少每周1次） 0＝否(**转问4.11. 饮茶**) 1＝是 | |  | |  | |
| 4.7.您习惯的饮酒方式为： | | |  | |  |
| 1=几乎天天喝，一般量少 | | |  | |  |
| 2=经常喝，且一般量大（半斤白酒以上） | | |  | |  |
| 3=不经常喝，但喝时一般量大（半斤白酒以上） | | |  | |  |
| 4=不经常喝，且一般量少 | | |  | |  |
| 4.8.您习惯饮酒的种类为： | | |  | |  |
| 1=啤酒 2=青稞酒 3=白酒 4=葡萄酒或果酒 5=黄酒 6=米酒 | | |  | | |
| 4.9.您饮酒共多少年? | | | 年 | |  |
| 4.10.您目前饮酒吗? 0＝否 1＝是 | | |  | |  |

|  | 4.10.1 您几年前戒酒的? | 年 | |
| --- | --- | --- | --- |
|  | 4.10.2您为何戒酒? 1=疾病 2=其它 |  | |
| 4.11. 您是否天天饮茶? 0=否（转问问题**4.14.**饮食方式）；1=是 | | |  |
| 4.12. 您本人平均每日饮茶的种类和饮茶量? | | |  |
| 1= 清茶 (砖茶+ 盐+ 水) | | | .升 |
| 2= 甜茶 （清茶+ 奶+红糖） | | | .升 |
| 3= 酥油茶 （清茶+ 酥油） | | | .升 |
| 4= 沏茶 （茶叶：红茶/绿茶/花茶 +水） 需加问4.13 | | | .升 |
| 5= 其他 请注明 | | | .升 |
| 4.13．您饮沏茶有多少年了？ | | | 年 |

4.14. 一般情况下，您食用下列食物的频率和食用量（必要时可请调查对象看表指出）：

|  | 1 | 2 | 3 | 4 | 5 |  |
| --- | --- | --- | --- | --- | --- | --- |
| 主食（糌粑） | 每日<5两 | 每日5-7两 | 每日8两-1斤 | 每日1.1-1.5斤 | 每日>1.5斤 |  |
| 主食（米/面） | 每日<5两 | 每日5-7两 | 每日8两-1斤 | 每日1.1-1.5斤 | 每日>1.5斤 |  |
| 豆制品（每斤豆浆折算为2两豆制品) | 每周<1两 | 每周1-4两 | 每周5-9两 | 每周1-1.5斤 | 每周>1.5斤 |  |
| 鸡、鸭等禽类肉 | 每月<5两 | 每月5-9两 | 每月1-1.9斤 | 每月2-3斤 | 每月>3斤 |  |
| 畜肉（包括猪、牛、羊等） | 每周<1两 | 每周1-4两 | 每周5-9两 | 每周1-1.5斤 | 每周>1.5斤 |  |
| 肥瘦程度(肥/瘦) | 瘦肉(0/10) | 较瘦(3/7) | 肥瘦(5/5) | 较肥(7/3) | 肥肉(10/0) |  |
| 鸡蛋、鸭蛋及其他蛋类 | 每周<1个 | 每周1-2个 | 每周3-5个 | 每周6-10个 | 每周>10个 |  |
| 奶及奶制品(每1勺奶粉折算为1两奶制品) | 每日<2两 | 每日2-4两 | 每日4-6两 | 每日6-10两 | 每日>1斤 |  |
| 新鲜蔬菜/水果（不包括土豆） | 每日<5两 | 每日5-7两 | 每日8两-1斤 | 每日1-1.5斤 | 每日>1.5斤 |  |
| 花生、瓜子、核桃等硬果 | 每周<1两 | 每周2-5两 | 每周6-9两 | 每周1-1.5斤 | 每周>1.5斤 |  |

**第五部分: 病史**

| 5.1.您患有高血压吗？0=无（转问Ⅱ 糖尿病） 1=有 9=不详（转问Ⅱ糖尿病） | | |  | |
| --- | --- | --- | --- | --- |
| 5.2 您的高血压病程有多少年? | | | 年 | |
| 5.3 您服用降压药的情况?  0=不服（转问5.6） 1=不舒服时服用 2=血压高时服用 3=吃完为止 4=坚持服 | | |  | |
| 5.4 您两周内服用降压药的情况? 0=不服　　　　 1=服 | | |  | |
| 5.5您服用的是藏药还是西药? 0=藏药； 1=西药 | | |  | |
| 5.6．您第一次诊断高血压是由谁诊断的? 1=藏医 2=西医 3=藏西医 4=不清楚 | | | |  |
| 5.7. 您患有糖尿病吗？ | | 0=无 1=有 9=不详  | | |
| 5.8.您患有脑卒中吗？ | | 0=无 1=有 9=不详  | | |
| 5.9. 您是否曾有过心肌梗死? | 0=无 1=有 9=不详  | | | |
| 5.10.曾经有医生诊断您患有其他疾病吗？ | 0=无 1=有 9=不详  | | | |

| 疾病名称 | 无 | 有 |  | 疾病名称 | 无 | | 有 | |  |
| --- | --- | --- | --- | --- | --- | --- | --- | --- | --- |
| 5.10.1.癌症 | 0 | 1 |  | 5.10.4.慢性呼吸系统疾病 | 0 | | 1 | |  |
| 5.10.2.肝脏疾病 | 0 | 1 |  | 5.10.5.高原性心脏病 | 0 | | 1 | |  |
| 5.10.3.肾脏疾病 | 0 | 1 |  | 5.10.6.痛风 | | 0 | | 1 |  |
| 5.10.7．其它慢性疾病： | | | | | |  | |  |  |

**第六部分: 家族史**

|  | 父亲 | 母亲 | 兄弟姐妹 |
| --- | --- | --- | --- |
| 6.1 高血压 |  |  |  |
| 6.2 冠心病 |  |  |  |
| 6.3 脑卒中 |  |  |  |
| 6.4 糖尿病 |  |  |  |
| 注：1.父母家族史请按“0＝无，1=有, 9＝不详”填写；  2.兄弟姐妹（不包括本人）无论几人阳性均填“1”，无一人阳性填“0”，不详填“9” ，无兄弟姐妹填“8”。 | | | |

**第七部分:　医疗资源调查**

| 7.1．当您感觉一般性的身体不适时，您会 | |  |
| --- | --- | --- |
| 1=随便吃点药 2=去村卫生所或镇卫生所 3=去县医院或更高一级医院  4=看藏医 5=不采取任何措施 | |  |
| 7.2．通常您不去就医的原因是 |  | |
| 1=医院距离远 2=就医费用贵 3=药品较少 4=其他_____________________ | | |

| 8.1.身高： | 厘米 |  | 8.2.体重: | . kg |
| --- | --- | --- | --- | --- |

**第八部分: 体格检查**

| 8.3．血压 | 第一次 / | 第二次 / |
| --- | --- | --- |
| 第三次 / | | |
| 8.4 第三次测量血压时心率: /分钟 | | |
